# Ca_v_1.2 regulates osteogenesis of bone marrow‐derived mesenchymal stem cells via canonical Wnt pathway in age‐related osteoporosis

**DOI:** 10.1111/acel.12967

**Published:** 2019-05-23

**Authors:** Dongdong Fei, Yang Zhang, Junjie Wu, Hui Zhang, Anqi Liu, Xiaoning He, Jinjin Wang, Bei Li, Qintao Wang, Yan Jin

**Affiliations:** ^1^ State Key Laboratory of Military Stomatology, Department of Periodontology, National Clinical Research Center for Oral Diseases, Shaanxi Engineering Research Center for Dental Materials and Advanced Manufacture School of Stomatology The Fourth Military Medical University Xi’an China; ^2^ State Key Laboratory of Military Stomatology, National Clinical Research Center for Oral Diseases, Shaanxi International Joint Research Center for Oral Diseases, Center for Tissue Engineering, School of Stomatology The Fourth Military Medical University Xi’an China; ^3^ Department of Orthopaedics, Xijing Hospital The Fourth Military Medical University Xi’an China; ^4^ State Key Laboratory of Military Stomatology, Department of Orthodontics, National Clinical Research Center for Oral Diseases, Shaanxi Clinical Research Center for Oral Diseases School of Stomatology The Fourth Military Medical University Xi’an China

**Keywords:** age‐related bone mass loss, bone marrow‐derived mesenchymal stem cells, osteogenic differentiation, voltage‐gated Ca^2+^ channels, Wnt/β‐catenin signaling, Zmpste24

## Abstract

**Aims:**

Age‐related bone mass loss is one of the most prevalent diseases that afflict the elderly population. The decline in the osteogenic differentiation capacity of bone marrow‐derived mesenchymal stem cells (BMMSCs) is regarded as one of the central mediators. Voltage‐gated Ca^2+^ channels (VGCCs) play an important role in the regulation of various cell biological functions, and disruption of VGCCs is associated with several age‐related cellular characteristics and systemic symptoms. However, whether and how VGCCs cause the decreased osteogenic differentiation abilities of BMMSCs have not been fully elucidated.

**Methods:**

Voltage‐gated Ca^2+^ channels related genes were screened, and the candidate gene was determined in several aging models. Functional role of determined channel on osteogenic differentiation of BMMSCs was investigated through gain and loss of function experiments. Molecular mechanism was explored, and intervention experiments in vivo and in vitro were performed.

**Results:**

We found that Ca_v_1.2 was downregulated in these aging models, and downregulation of Ca_v_1.2 in Zmpste24−/− BMMSCs contributed to compromised osteogenic capacity. Mechanistically, Ca_v_1.2 regulated the osteogenesis of BMMSCs through canonical Wnt/β‐catenin pathway. Moreover, upregulating the activity of Ca_v_1.2 mitigated osteoporosis symptom in Zmpste24−/− mice.

**Conclusion:**

Impaired osteogenic differentiation of Zmpste24−/− BMMSCs can be partly attributed to the decreased Ca_v_1.2 expression, which leads to the inhibition of canonical Wnt pathway. Bay K8644 treatment could be an applicable approach for treating age‐related bone loss by ameliorating compromised osteogenic differentiation capacity through targeting Ca_v_1.2 channel.

**Abbreviations**
ALP: alkaline phosphatase
BMD: bone mineral density
BMMSCs: bone marrow‐derived mesenchymal stem cells
BV/TV: bone volume/total volume
MAR: mineral apposition rate
OCN: osteocalcin
qRT–PCR: quantitative real‐time polymerase chain reaction
Runx2: runt‐related transcription factor 2
SAMP6: senescence‐accelerated mouse prone 6
SAMR1: senescence‐accelerated mice‐resistant 1
Tb.N: trabecular number
VGCCs: voltage‐gated Ca^2+ ^channels

## 1 INTRODUCTION

Aging is a progressive deterioration of physiological functions accompanied by bone loss, leading to bone fragility, a disease that is known as osteoporosis (Singh, Brennan, et al., 2016). With advancing age, the balance between bone formation and resorption is compromised, resulting in overall bone loss and structural damage. It has been confirmed that the impaired capacity of osteogenic differentiation of BMMSCs from the elderly people is a causative factor leading to age‐related osteoporosis (Baker, Boyette, & Tuan, 2015). However, the underlying mechanisms that account for impaired osteogenic differentiation of aging BMMSCs remain unknown.

Calcium channels, which are in charge of calcium transportation into and out of cells and organelles, play an important role in aging (Surmeier, 2007; Warnier et al., 2018). In terms of bone aging, numerous studies have indicated the relationship between disruption of calcium channels and age‐related bone mass loss (Agacayak et al., 2014; Shimizu et al., 2012). There are several kinds of calcium channels, among which voltage‐gated Ca^2+^ channels (VGCCs) are important components. They are formed by α1, α2δ, β, and γ subunits and are transmembrane surface proteins indispensable for many physiological events including membrane depolarizes, cellular motility, gene expression, and differentiation (Zamponi, Striessnig, Koschak, & Dolphin, 2015). On the basis of the differences in α1 subunits, there are three families of VGCCs in mammals known as Ca_v_1, Ca_v_2, and Ca_v_3 (Zamponi, 2016). Ca_v_1, also termed as L‐type calcium channel, can be further subdivided into Ca_v_1.1, Ca_v_1.2, Ca_v_1.3, and Ca_v_1.4. Similarly, Ca_v_2 can be classified into Ca_v_2.1, Ca_v_2.2, and Ca_v_2.3, and Ca_v_3 encompasses Ca_v_3.1, Ca_v_3.2, and Ca_v_3.3. Abnormal expressions of VGCCs have been linked with several age‐related cellular characteristics and systemic symptoms (Silva et al., 2017; Zamponi, 2016). Previous study confirmed that age‐associated downregulation of Ca_v_3.1 was involved in increased production of amyloid beta peptide 1–42 in N2a cells that led to neurodegenerative diseases (Rice, Berchtold, Cotman, & Green, 2014). However, unlike other age‐related diseases, the relationship between VGCCs and age‐related bone loss remains a great dispute. Several studies showed that the activation of VGCCs played an important role in bone formation (Li, Duncan, Burr, Gattone, & Turner, 2003; Li, Duncan, Burr, & Turner, 2002; Noh, Park, Zheng, Ha, & Yim, 2011), while others reported that inhibition of VGCCs could promote bone formation and suppress bone resorption (Marie, 2010; Ritchie, Maercklein, & Fitzpatrick, 1994). Besides, whether and how VGCCs cause the decreased osteogenic differentiation of BMMSCs is poorly explored at the molecular level.

It has been widely acknowledged that canonical Wnt pathway plays a significant role in the regulation of BMMSCs osteogenic differentiation (Cook, Fellgett, Pownall, O'Shea, & Genever, 2014; Lin & Hankenson, 2011). The canonical Wnt pathway causes an accumulation of β‐catenin in the cytoplasm and its eventual translocation into the nucleus to act as a transcriptional coactivator. In Wnt signaling, GSK3β, a negative regulator of the canonical Wnt signaling, forms a multimeric complex with APC, AXIN1, and β‐catenin and leads to β‐catenin degradation by targeting it for ubiquitination. Phosphorylation of GSK3β can interfere with the complex and block β‐catenin degradation. Numerous studies have confirmed that inhibition of Wnt/β‐catenin antagonists, such as sclerostin, DKK1, and WIF‐1, can emerge as a promising therapeutic approach in the treatment of osteoporosis, and some are even in Phase III studies (Chen et al., 2014; Ke, Richards, Li, & Ominsky, 2012). However, it remains unknown whether VGCCs could regulate osteogenic differentiation through canonical Wnt pathway.

Zmpste24 is a metalloproteinase that partakes in the maturation of lamin A. Zmpste24‐deficient mice recapitulate many phenotypes observed in physiological aging, including reduced bone density and an increased risk of fracture (Ghosh & Zhou, 2014). The availability of Zmpste24‐deficient mice could provide a new model to aid the study of mechanistic events underlying age‐related osteoporosis. In this study, we provide insights into the importance of VGCCs in regulating age‐related bone mass loss and the mechanism that underlie VGCCs mediated bone mass loss in Zmpste24‐deficient mice. We screened the expression of VGCCs in BMMSCs from Zmpste24‐deficient mice and natural aging mice models and found that *Ca_v_1.2* was downregulated. Downregulation of Ca_v_1.2 was responsible for defective osteogenic differentiation of aging BMMSCs. Mechanistically, Ca_v_1.2 regulated the osteogenesis of BMMSCs through Wnt/β‐catenin pathway. Moreover, activating Ca_v_1.2 channel mitigated osteoporosis symptom in Zmpste24−/− mice.

## 2 RESULTS

### 2.1 Ca_v_1.2 is downregulated in aging BMMSCs with impaired osteogenic differentiation

**Figure 1:**
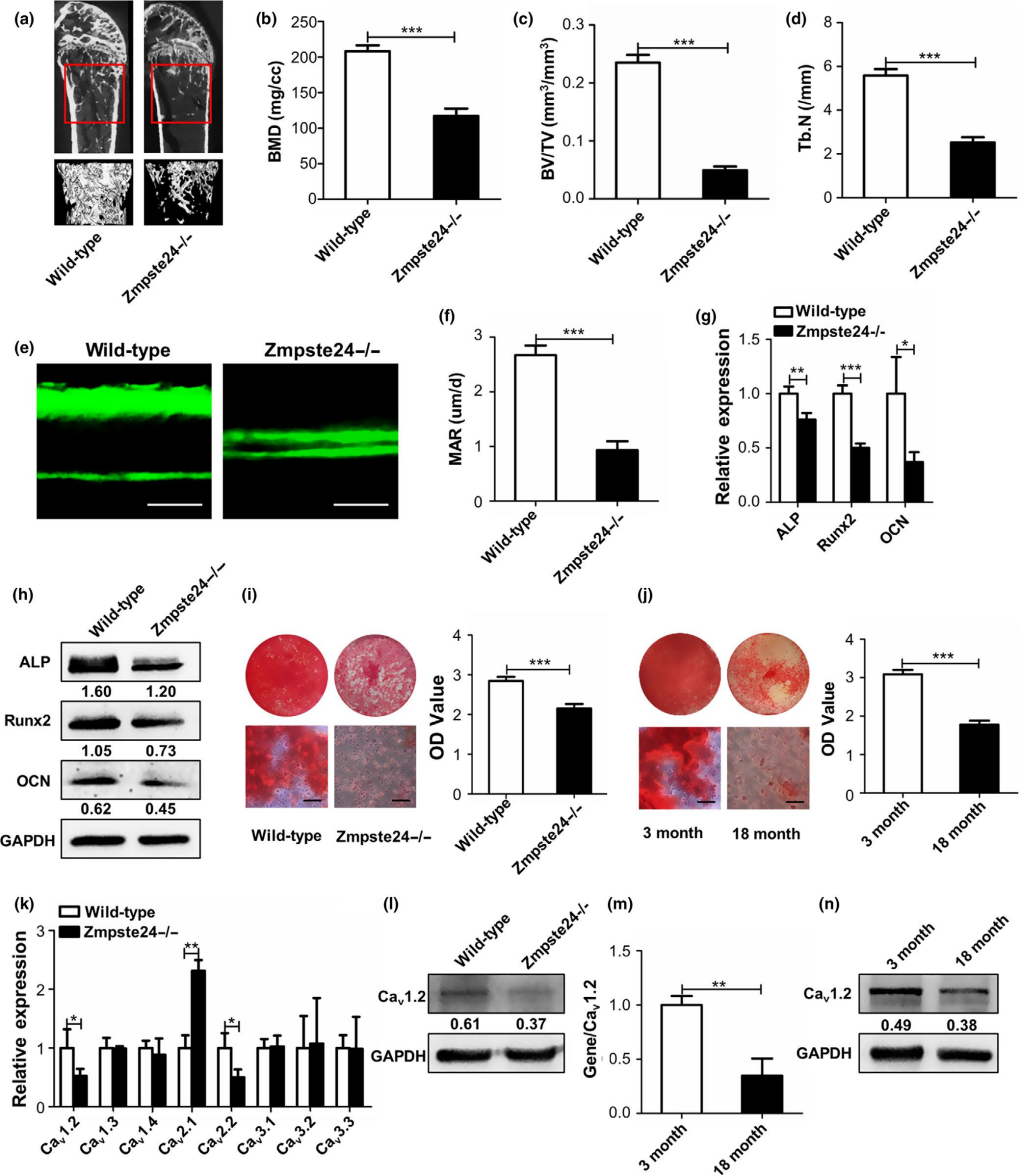
Ca_v_1.2 is downregulated in aging BMMSCs with impaired osteogenic differentiation. (a) Bone masses of 3‐month‐old wild‐type and Zmpste24‐deficient mice were tested by micro‐CT (*n* = 3), and the “interesting zone” was highlighted. (b–d) Bone mineral density (BMD), bone volume/total volume (BV/TV), and trabecular number (Tb.N) of 3‐month‐old wild‐type and Zmpste24‐deficient mice were analyzed with the micview software. (e) Bone formations of 3‐month‐old wild‐type and Zmpste24−/− mice were examined by double‐calcein labeling (*n* = 3). Scale bar, 25 um. (f) Mineral apposition rate (MAR) was analyzed by Image J under fluorescence microscope. (g) Expressions of osteogenic‐related genes of *ALP*, *Runx2,* and *OCN* in wild‐type and Zmpste24‐deficient mice were detected by qRT–PCR after osteogenic induction for 5 days (*n* = 3). The results were normalized to GAPDH. (h) Expressions of osteogenic‐related proteins of ALP, Runx2, and OCN in wild‐type and Zmpste24‐deficient mice were detected by Western blot after osteogenic induction for 7 days (*n* = 3). GAPDH was used as an internal control. (i) Mineralized nodules of BMMSCs from wild‐type and Zmpste24‐deficient mice were assayed by alizarin red staining after osteogenic induction for 14 days and quantified with a spectrophotometer after dissolving with cetylpyridinium chloride (*n* = 3). (j) Alizarin red staining was performed to detect mineralized nodules of BMMSCs from 3‐month‐old to 18‐month‐old normal mice after osteogenic induction for 14 days and quantified with a spectrophotometer after dissolving with cetylpyridinium chloride (*n* = 3). (k) Expressions of voltage‐gated Ca^2+ ^channels (VGCCs) related genes in 3‐month‐old wild‐type and Zmpste24‐deficient mice were explored by qRT–PCR (*n* = 7). The results were normalized to GAPDH. (l) The protein level of Ca_v_1.2 in 3‐month‐old wild‐type and Zmpste24‐deficient mice was explored by Western blot (*n* = 7). (m) Expression of *Ca_v_1.2* in 3‐month‐old young mice and 18‐month‐old natural aging mice was explored by qRT–PCR (*n* = 7). The results were normalized to GAPDH. (n) The protein level of Ca_v_1.2 in 3‐month‐old young mice and 18‐month‐old natural aging mice was explored by Western blot (*n* = 7). Scale bar of alizarin red, 50 um. Data are shown as mean ± *SD*. *p < 0.05, **p < 0.01, ***p < 0.001, which was determined by unpaired two‐tailed Student's *t* test

Genotype identification of wild‐type, Zmpste24+/‐, and Zmpste24−/− mice was shown in Figure S1. Firstly, we used micro‐CT to measure relative density of bone mineral from 3‐month‐old wild‐type and Zmpste24‐deficient mice, and micro‐CT images showed reduced mineralization of Zmpste24‐deficient mice (Figure 1a). Bone mineral density (BMD), bone volume/total volume (BV/TV), and trabecular number (Tb.N) were also significantly decreased in Zmpste24‐deficient mice as measured by micro‐CT densitometry (Figure 1b–d). Besides, bone formations were also significantly decreased as carried out by double‐calcein labeling (Figure 1e,f). Given that the osteogenic differentiation ability of BMMSCs is closely related to osteogenesis, we further detected alteration of osteogenic differentiation of BMMSCs and found the expressions of osteogenic‐related genes and proteins, ALP, Runx2, and OCN were downregulated in Zmpste24−/− BMMSCs after osteogenic induction as assayed by qRT–PCR and Western blot (Figure 1g,h). Alizarin red staining also showed less mineralized nodules formation in Zmpste24−/− BMMSCs than wild‐type BMMSCs (Figure 1i), which is consistent with the result from BMMSCs that derived from 3‐month‐old young mice and 18‐month‐old natural aging mice (Figure 1j). To investigate whether VGCCs are involved in the regulation of osteogenic differentiation of BMMSCs during aging process, qRT–PCR analysis was performed to measure the expressions of VGCCs related genes in several aging models. The results showed that lower expressions of *Ca_v_1.2* and *Ca_v_2.2* in BMMSCs from Zmpste24‐deficient mice compared to the wild‐type mice (Figure 1k). We also screened the expressions of VGCCs related genes in 3‐month‐old SAMR1 mice and SAMP6 mice. The results showed that *Ca_v_1.2* and osteogenesis were still downregulated in BMMSCs from 3‐month‐old SAMR1 mice compared with SAMP6 mice (Figure S2a,b). Besides, we also explored expressions of VGCCs related genes in BMMSCs from young individuals compared with that from old individuals (donor information was listed in Table S1) and found changes of VGCCs associated genes, among which *Ca_v_1.2* was still downregulated (Figure S3a,b). Moreover, we also confirmed downregulated *Ca_v_1.2* expression of BMMSCs in natural aging model (Figure 1m). To further confirm the change of Ca_v_1.2 during aging process, we also investigated the protein level of Ca_v_1.2. Western blot analysis showed that Ca_v_1.2 protein was also downregulated in Zmpste24−/− and natural aging mice (Figure 1l,n).

### 2.2 Ca_v_1.2 regulates osteogenic differentiation of aging BMMSCs

**Figure 2:**
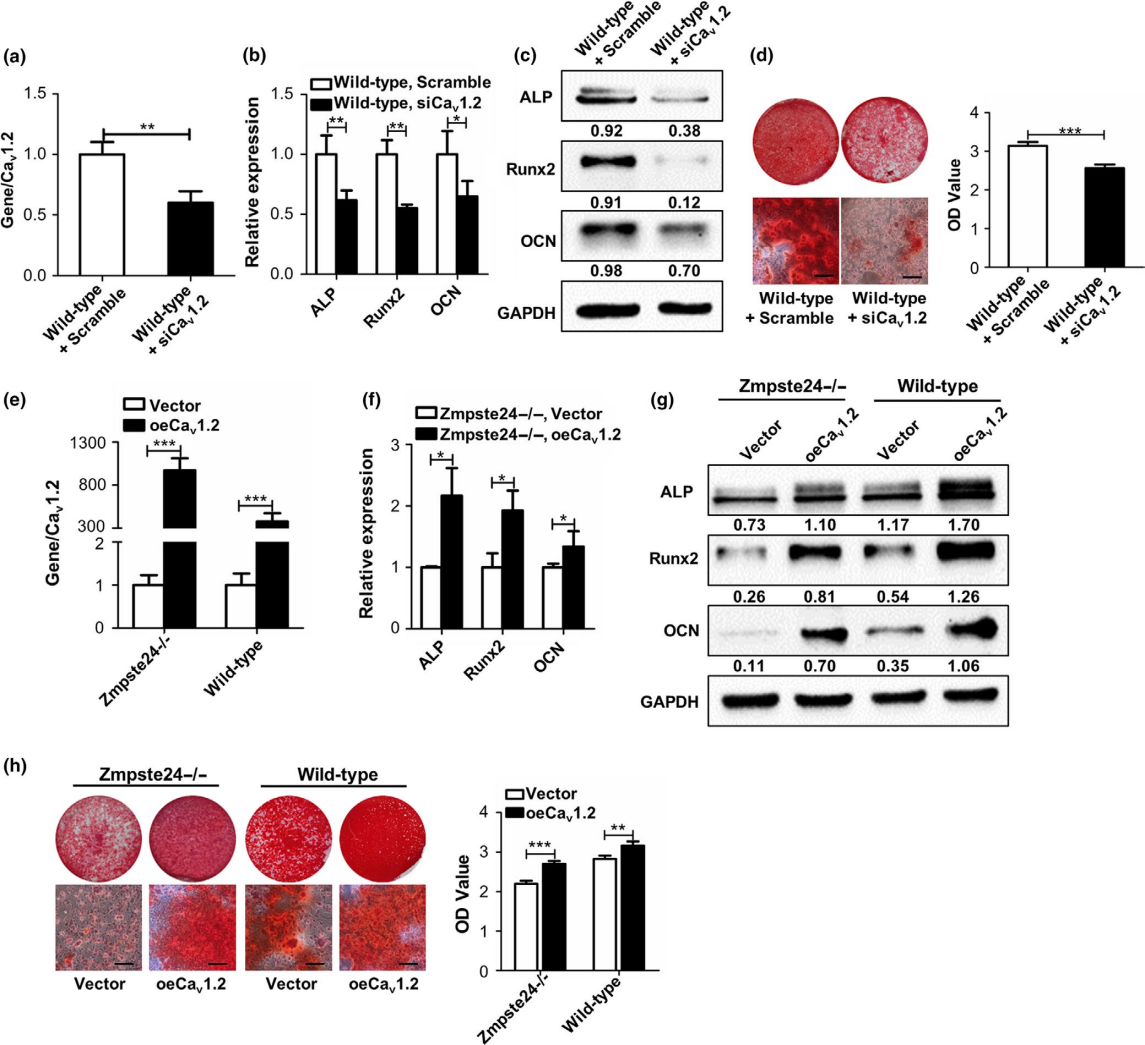
Ca_v_1.2 regulates osteogenic differentiation of aging BMMSCs. (a) Wild‐type BMMSCs were transfected with scramble siRNA or Ca_v_1.2 siRNA, and the transfection efficiency was tested by qRT–PCR after transfection for 48 hr (*n* = 3). (b) Expressions of osteogenic‐related genes of *ALP*, *Runx2,* and *OCN* in wild‐type BMMSCs transfected with scramble siRNA or Ca_v_1.2 siRNA were detected by qRT–PCR after osteogenic induction for 5 days (*n* = 3). (c) Expressions of osteogenic‐related proteins of ALP, Runx2, and OCN in wild‐type BMMSCs transfected with scramble siRNA or Ca_v_1.2 siRNA were detected by Western blot after osteogenic induction for 7 days (*n* = 3). (d) Alizarin red staining of wild‐type BMMSCs transfected with scramble siRNA or Ca_v_1.2 siRNA were performed after osteogenic induction for 14 days (*n* = 3). (e) Wild‐type and Zmpste24−/− BMMSCs were transfected with control overexpression vector or Ca_v_1.2 plasmid, and the transfection efficiency was tested by qRT–PCR after transfection for 48 hr (*n* = 3). (f) Wild‐type and Zmpste24−/− BMMSCs were transfected with control overexpression vector or Ca_v_1.2 plasmid, and the expressions of osteogenic‐related genes of *ALP*, *Runx2,* and *OCN* were detected by qRT–PCR after osteogenic induction for 5 days (*n* = 3). (g) Wild‐type and Zmpste24−/− BMMSCs were transfected with control overexpression vector or Ca_v_1.2 plasmid, and the expressions of osteogenic‐related proteins of ALP, Runx2, and OCN were detected by Western blot after osteogenic induction for 7 days (*n* = 3). (h) Wild‐type and Zmpste24−/− BMMSCs were transfected with control overexpression vector or Ca_v_1.2 plasmid, and alizarin red staining was performed after osteogenic induction for 14 days (*n* = 3). The expression levels of the target genes and proteins were normalized to GAPDH. Scale bar, 50 um. Data are shown as mean ± *SD*. *p < 0.05, **p < 0.01, ***p < 0.001, which was determined by paired two‐tailed Student's *t* test

With the aim of investigating the potential role of Ca_v_1.2 on impaired osteogenic differentiation of Zmpste24−/− BMMSCs, we modulated Ca_v_1.2 expression levels in both wild‐type and Zmpste24−/− BMMSCs. Transfection of Ca_v_1.2 siRNA into wild‐type BMMSCs decreased Ca_v_1.2 levels (Figure 2a), while overexpression of Ca_v_1.2 led to the upregulation of Ca_v_1.2 expression levels (Figure 2e). The results showed that decline of Ca_v_1.2 expression levels in wild‐type BMMSCs decreased the expressions of osteogenic differentiation‐related genes and proteins of ALP, Runx2, and OCN after osteogenic induction (Figure 2b,c). After knockdown of Ca_v_1.2, formation of mineralized nodules was also reduced (Figure 2d). We also overexpressed Ca_v_1.2 in wild‐type and Zmpste24−/− BMMSCs by transfection of Ca_v_1.2 overexpression vector. Overexpression of Ca_v_1.2 enhanced the osteogenic differentiation of wild‐type and Zmpste24−/− BMMSCs, as Ca_v_1.2 overexpression groups exhibited higher expressions of ALP, Runx2, and OCN in gene and protein levels after osteogenic induction (Figure 2f,g), and more mineralized nodules were also confirmed as measured by quantitative analysis (Figure 2h).

We also investigated the effect of Ca_v_1.2 on adipogenic differentiation as BMMSCs exhibit an age‐dependent reduction in osteogenic differentiation with an increased tendency toward adipocyte differentiation (Chen et al., 2016). Our results also confirmed the increased adipogenic differentiation ability of Zmpste24−/− BMMSCs (Figure S4a). To further explore the effect of Ca_v_1.2 on adipogenic differentiation ability of Zmpste24−/− BMMSCs, we overexpressed Ca_v_1.2 in Zmpste24−/− BMMSCs and no significant difference of adipogenic differentiation was found compared to Zmpste24−/− BMMSCs (Figure S4b).

### 2.3 Ca_v_1.2 upregulates osteogenic differentiation of BMMSCs via Wnt/β‐catenin pathway

**Figure 3:**
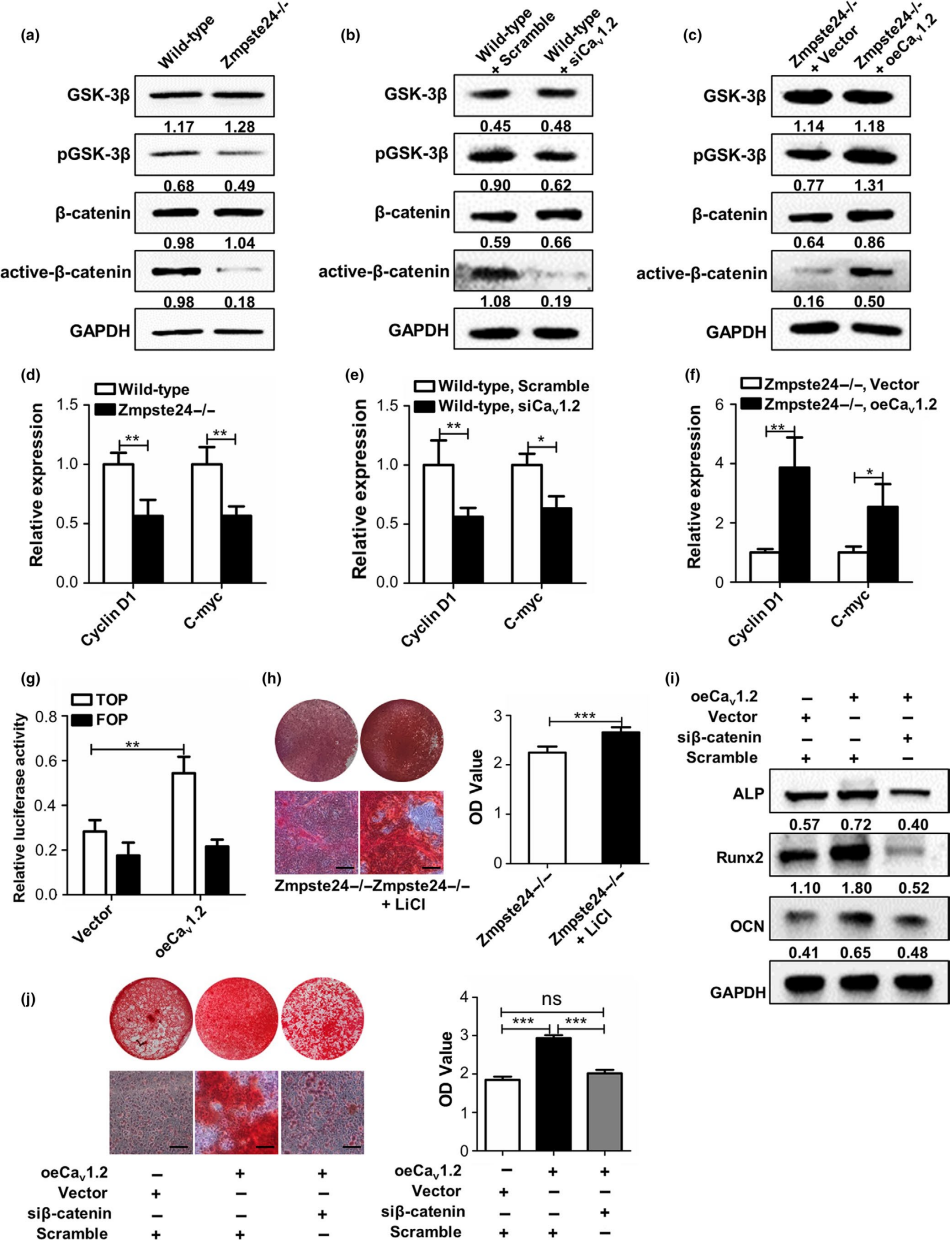
Ca_v_1.2 upregulates osteogenic differentiation of BMMSCs via Wnt/β‐catenin pathway. (a) Expressions of GSK3β, p‐GSK3β, β‐catenin, and active‐β‐catenin in wild‐type and Zmpste24−/− BMMSCs were detected by Western blot analysis (*n* = 7). (b) Wild‐type BMMSCs were transfected with scramble siRNA or Ca_v_1.2 siRNA, and the protein expression levels of GSK3β, p‐GSK3β, β‐catenin, and active‐β‐catenin were confirmed by Western blot analysis after 72 hr (*n* = 3). (c) Zmpste24−/− BMMSCs were transfected with control overexpression vector or Ca_v_1.2 plasmid, and the protein expression levels of GSK3β, p‐GSK3β, β‐catenin, and active‐β‐catenin were confirmed by Western blot analysis after 72 hr (*n* = 3). (d) Wnt target genes of cyclin D1 and c‐myc were explored in wild‐type and Zmpste24−/− BMMSCs by qRT–PCR (*n* = 7). (e) Wild‐type BMMSCs were transfected with scramble siRNA or Ca_v_1.2 siRNA, and Wnt target genes of cyclin D1 and c‐myc were explored by qRT–PCR (*n* = 3). (f) Zmpste24−/− BMMSCs were transfected with control overexpression vector or Ca_v_1.2 plasmid, and Wnt target genes of cyclin D1 and c‐myc were explored by qRT–PCR (*n* = 3). (g) 48 hr after transfection with control overexpression vector or Ca_v_1.2 plasmid in progerin‐overexpressed 293T cells, TOP/FOP, and Renilla luciferase plasmid were transfected, and TOP/FOP reporter assay was performed to detect the activity of β‐catenin after 24 hr (*n* = 3). (h) Alizarin red staining of Zmpste24−/− and 5 mM LiCl‐treated Zmpste24−/− BMMSCs was performed to detect mineralized nodules after osteogenic induction for 14 days (*n* = 3). (i) Expressions of osteogenic‐related proteins of ALP, Runx2, and OCN in Zmpste24−/− BMMSCs from control group, Ca_v_1.2‐overexpressed and Ca_v_1.2‐overexpressed in the context of β‐catenin siRNA were detected by Western blot after osteogenic induction for 7 days (*n* = 3), and each group contains equal volume of transfection agent. (j) Alizarin red staining of Zmpste24−/− BMMSCs from control group, Ca_v_1.2‐overexpressed and Ca_v_1.2‐overexpressed in the context of β‐catenin siRNA were performed after osteogenic induction for 14 days (*n* = 3), and each group contains equal volume of transfection agent. The expression levels of the target proteins were normalized to GAPDH. Scale bar, 50 um. Data are shown as mean ± *SD*. *p < 0.05, **p < 0.01, ***p < 0.001, which was determined by paired two‐tailed Student's *t* test

Given that canonical Wnt/β‐catenin signaling pathway plays an essential role in regulating osteogenic differentiation of BMMSCs, we speculated that Ca_v_1.2 regulated osteogenic differentiation via canonical Wnt/β‐catenin signaling. In order to confirm our hypothesis, we first detected canonical Wnt/β‐catenin pathway in wild‐type and Zmpste24−/− BMMSCs to determine whether the canonical Wnt/β‐catenin signaling is disrupted in Zmpste24−/− BMMSCs. Western blot analysis revealed decreased expressions of p‐GSK3β and active‐β‐catenin in Zmpste24−/− BMMSCs (Figure 3a). QRT–PCR analysis also showed less expressions of Wnt target genes of cyclin D1 and c‐myc in Zmpste24−/− BMMSCs (Figure 3d). Then, we investigated whether abnormity of Wnt/β‐catenin pathway in Zmpste24−/− BMMSCs contributed to impaired osteogenic differentiation by using Wnt/β‐catenin activator lithium chloride (LiCl), which can activate Wnt/β‐catenin pathway through inhibiting GSK3β (Clement‐Lacroix et al., 2005). Zmpste24−/− BMMSCs were incubated with 5 mM LiCl, as this concentration could significantly activate Wnt/β‐catenin pathway (Figure S5a,b). The Alizarin red staining analysis verified enhanced calcified nodule forming in Zmpste24−/− BMMSCs after LiCl stimulation (Figure 3h). All these data indicated that inhibited canonical Wnt/β‐catenin pathway led to defective osteogenic differentiation of Zmpste24−/− BMMSCs.

To investigate whether Ca_v_1.2 regulated osteogenic differentiation through Wnt/β‐catenin signaling, we used siRNA to knockdown Ca_v_1.2 in wild‐type BMMSCs. The results showed that both p‐GSK3β and active‐β‐catenin expressions were decreased after knockdown of Ca_v_1.2 (Figure 3b). Moreover, overexpressing Ca_v_1.2 in Zmpste24−/− BMMSCs presented increased expressions of p‐GSK3β and active‐β‐catenin (Figure 3c). Besides, Wnt target genes of cyclin D1 and c‐myc were also downregulated after knockdown of Ca_v_1.2 in wild‐type BMMSCs and upregulated after overexpression of Ca_v_1.2 in Zmpste24−/− BMMSCs (Figure 3e,f). Next, we employed TOP/FOP flash luciferase reporter assay to confirm enhanced Wnt signaling after overexpressing Ca_v_1.2 in progerin‐overexpressed 293T cells. The results also showed a significant increase in β‐catenin activity after overexpressing Ca_v_1.2 (Figure 3g). To further verify the involvement of Wnt/β‐catenin signaling for Ca_v_1.2‐mediated osteogenic differentiation, we overexpressed Ca_v_1.2 in Zmpste24−/− BMMSCs in the context of β‐catenin siRNA followed by analysis of the osteogenic differentiation. The results further confirmed that canonical Wnt signaling pathway is involved in Ca_v_1.2‐mediated osteogenic differentiation regulation (Figure 3i,j). We also investigated the other two signal pathways, PKC‐ERK1/2 (Muchekehu & Harvey, 2008; Soletti et al., 2010) and CAMKII (Liu et al., 2018; Wei, Wang, Wang, & Bai, 2017), as they can also be regulated by calcium channel. We found that the expression of Ca_v_1.2 has no significant effect on these two signal pathways after inhibiting Ca_v_1.2 in wild‐type BMMSCs (Figure S6a) and overexpressing Ca_v_1.2 in Zmpste24−/− BMMSCs (Figure S6b), which further verified that Ca_v_1.2‐mediated osteogenic differentiation was through Wnt/β‐catenin pathway.

### 2.4 Bay K8644 treatment activates Ca_v_1.2 and rescues the impaired osteogenic differentiation of ZMPSTe24−/− BMMSCs

**Figure 4:**
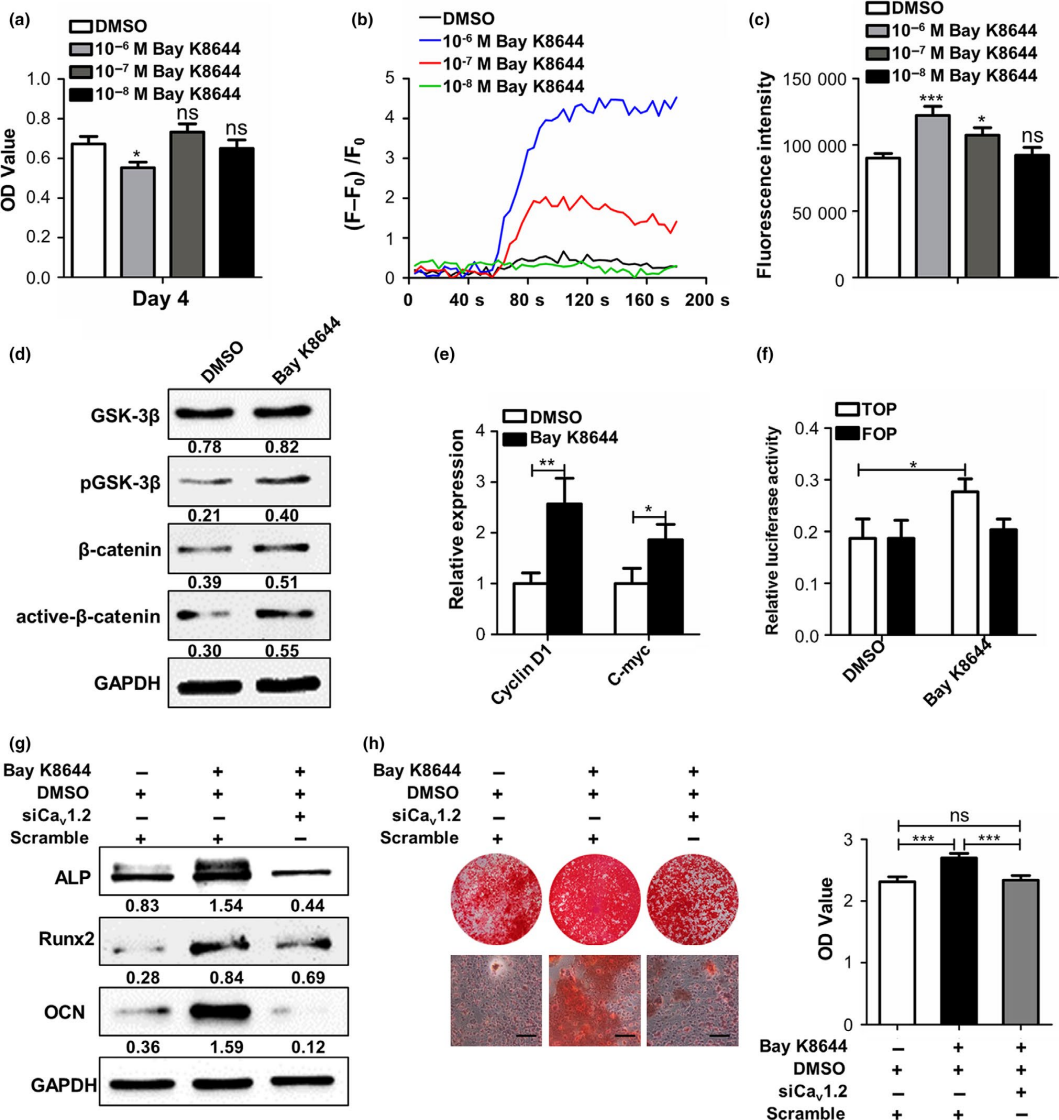
Bay K8644 treatment activates Ca_v_1.2 and rescues the impaired osteogenic differentiation of Zmpste24−/− BMMSCs. (a) After treated by DMSO or 10^‐6^, 10^‐7^, and 10^‐8 ^M Bay K8644 for 3 days, the proliferation of Zmpste24−/− BMMSCs was detected by MTT (*n* = 3). (b) After treated by DMSO or 10^‐6^, 10^‐7^, and 10^‐8 ^M Bay K8644 for 3 days, intracellular calcium current of Zmpste24−/− BMMSCs was explored by laser confocal microscopy (*n* = 3). (c) After treated by DMSO or 10^‐6^, 10^‐7^, and 10^‐8 ^M Bay K8644 for 3 days, intracellular calcium concentration of Zmpste24−/− BMMSCs was explored by flow cytometry (*n* = 3). (d) After treated by 10^‐7^ M Bay K8644 or DMSO for 3 days, the protein expression levels of GSK3β, p‐GSK3β, β‐catenin, and active‐β‐catenin in Zmpste24−/− BMMSCs were confirmed by Western blot analysis (*n* = 3). (e) After treated by 10^‐7^ M Bay K8644 or DMSO for 3 days, Wnt target genes of cyclin D1 and c‐myc were explored by qRT–PCR (*n* = 3). (f) 48 hr after stimulated by DMSO or Bay K8644 in progerin‐overexpressed 293T cells, TOP/FOP, and Renilla luciferase plasmid were transfected, and TOP/FOP reporter assay was performed to detect the activity of β‐catenin after 24 hr (*n* = 3). (g) After osteogenic induction for 7 days, expressions of osteogenic‐related proteins of ALP, Runx2, and OCN in Zmpste24−/− BMMSCs of DMSO‐treated, Bay K8644‐treated, and Bay K8644‐treated in the context of Ca_v_1.2 siRNA were explored (*n* = 3). (h) After osteogenic induction for 14 days, mineralized nodule formations in Zmpste24−/− BMMSCs of DMSO‐treated, Bay K8644‐treated and Bay K8644‐treated in the context of Ca_v_1.2 siRNA were explored (*n* = 3). The expression levels of the target genes and proteins were normalized to GAPDH. Scale bar, 50 um. Data are shown as mean ± *SD*. *p < 0.05, **p < 0.01, ***p < 0.001, which was determined by paired two‐tailed Student's *t* test

The notion that reduced Ca_v_1.2 expression contributes to compromised osteogenic differentiation of Zmpste24−/− BMMSCs suggested that it might be possible to rescue the differentiation through activating channels pharmacologically. Considering that Ca_v_1.2 belongs to L‐type calcium channel, we investigated this possibility by using the potent L‐type Ca^2+^ channel agonist Bay K8644. Firstly, we determined the optimal concentration of Bay K8644 by measuring cell proliferation, intracellular calcium current, and calcium concentration. We determined the final concentration of Bay K8644 at 10^‐7^ M as this concentration could significantly elevate intracellular calcium current and calcium concentration without inhibiting the proliferation of cells (Figure 4a–c). Besides, as supported by increased expressions of p‐GSK3β and active‐β‐catenin, remarkable activation of Wnt/β‐catenin pathway of Zmpste24−/− BMMSCs was noticed after stimulated by 10^−7^ M Bay K8644 (Figure 4d). QRT–PCR also showed enhanced expressions of Wnt target genes of cyclin D1 and c‐myc after 10^−7^ M Bay K8644 treatment (Figure 4e). Besides, results from TOP/FOP flash luciferase reporter assay that performed in progerin‐overexpressed 293T cells also showed enhanced Wnt signaling after Bay K8644 treatment (Figure 4f). Moreover, 10^−7^ M Bay K8644 treatment for 7 days promoted expressions of osteogenic proteins in Zmpste24−/− BMMSCs after osteogenic induction (Figure 4g). Alizarin red assays showed more mineral node formation in Bay K8644‐treated groups (Figure 4h). We also inhibited the expression of Ca_v_1.2 in Zmpste24−/− BMMSCs during Bay K8644 treatment followed by analysis of the osteogenic differentiation, and the results showed that activation of Ca_v_1.2 was indispensable for Bay K8644‐mediated osteogenic differentiation improvement (Figure 4g,h). To sum up, these results suggested that Bay K8644 rescued osteogenic differentiation of Zmpste24−/− BMMSCs through Ca_v_1.2.

### 2.5 Intraperitoneal injection of Bay K8644 mitigates osteoporosis symptom in Zmpste24−/− mice

**Figure 5:**
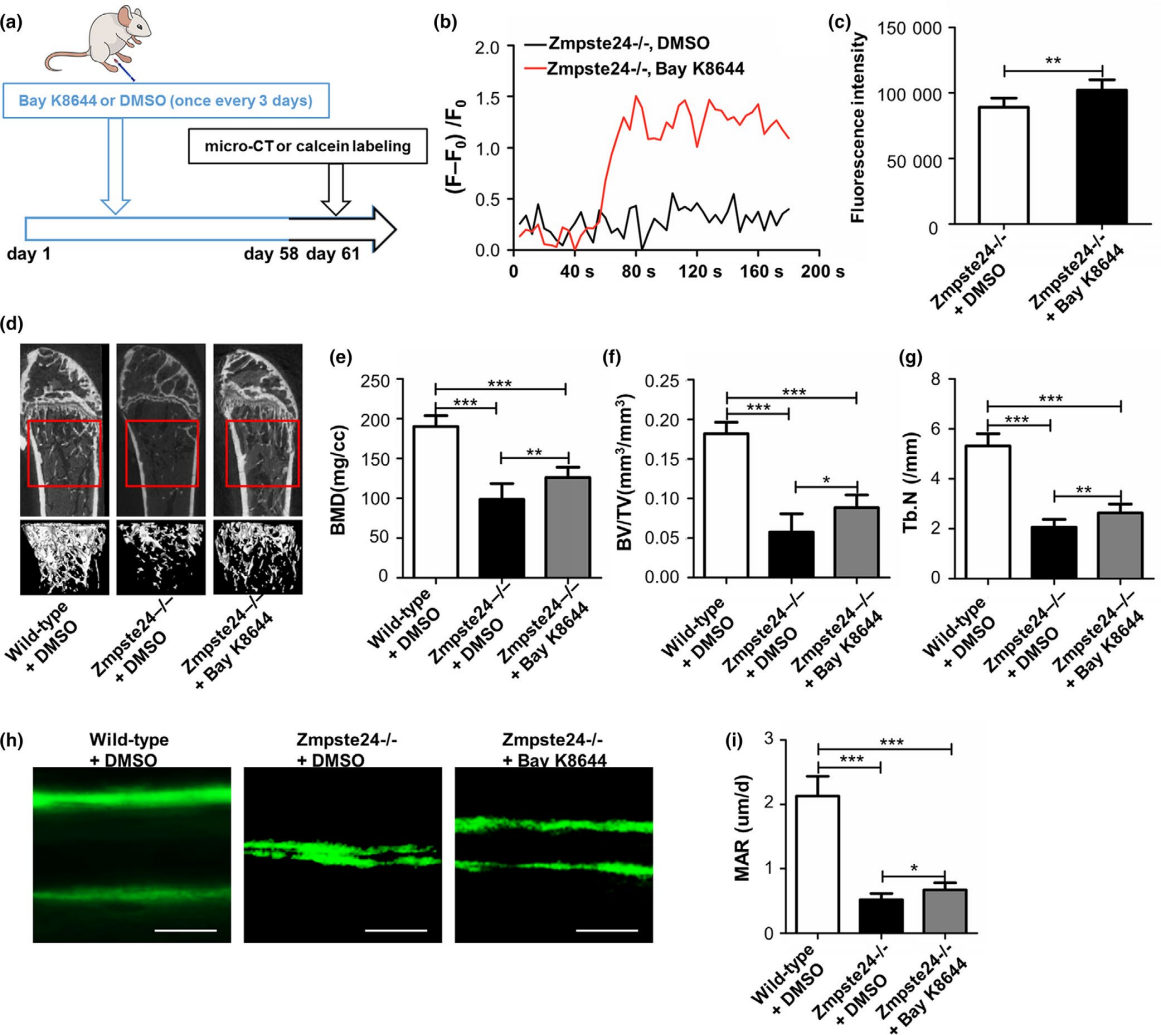
Intraperitoneal injection of Bay K8644 mitigates osteoporosis symptom of Zmpste24−/− mice. (a) The scheme for intraperitoneal injection and subsequent examination were shown. (b) After intraperitoneal injection for 5 days, BMMSCs from DMSO‐treated and Bay K8644‐treated Zmpste24−/− mice were isolated, and intracellular calcium currents were tested by laser confocal microscopy (*n* = 7). (c) After intraperitoneal injection for 5 days, BMMSCs from DMSO‐treated and Bay K8644‐treated Zmpste24−/− mice were isolated, and intracellular calcium concentrations were tested by flow cytometry assay (*n* = 7). (d) After intraperitoneal injection for 2 months, bone masses of DMSO‐treated wild‐type, DMSO‐treated Zmpste24−/−, and Bay K8644‐treated Zmpste24−/− groups were tested by micro‐CT (*n* = 6, 7 and 7). The “interesting zone” was highlighted. (e–g) BMD, BV/TV, and Tb.N were analyzed with the micview software (*n* = 6, 7 and 7). (h) After intraperitoneal injection for 2 months, bone formations of DMSO‐treated wild‐type, DMSO‐treated Zmpste24−/−, and Bay K8644‐treated Zmpste24−/− groups were examined by double‐calcein labeling (*n* = 6, 7 and 7). Scale bar, 25 um. (i) Mineral apposition rate (MAR) was analyzed by Image J under fluorescence microscope. Data are shown as mean ± *SD*. *p < 0.05, **p < 0.01, ***p < 0.001, which was determined by unpaired two‐tailed Student's *t* test

To investigate whether intraperitoneal injection of Bay K8644 could alleviate osteoporosis symptom of Zmpste24−/− mice, 1 mg/kg Bay K8644 was administrated to 3‐month‐old Zmpste24−/− mice through intraperitoneal injection. The scheme for intraperitoneal injection was shown in Figure 5a. The results showed that this dosage treatment could improve intracellular calcium current and calcium concentration of Zmpste24−/− BMMSCs after 5 days stimulation (Figure 5b,c). We also set the groups of DMSO‐treated Zmpste24−/− and DMSO‐treated wild‐type mice. The treatment was initiated when male mice at 3 months of age and was given every 3 days. After treating for two months, mice were sacrificed for Micro‐CT analysis or processed to perform calcein labeling assay. As shown in Figure 5d–g, BMD, BV/TV, and Tb.N were significantly increased in Bay K8644‐treated Zmpste24−/− mice compared to DMSO‐treated Zmpste24−/− mice. In addition, Bay K8644 treatment also significantly enhanced bone formation as carried out by double‐calcein labeling (Figure 5h,i). However, we also found that BMD, BV/TV, and Tb.N were still lower compared with DMSO‐treated wild‐type group (Figure 5d–g), which indicated that Bay K8644 could only partly ameliorate osteoporosis symptom. Consistent with such observations, double‐calcein labeling also implied partial recovery of osteoporosis features after Bay K8644 treatment (Figure 5h,i).

### 2.6 Bay K8644 rescues osteogenic differentiation ability of Zmpste24−/− BMMSCs in vivo

**Figure 6:**
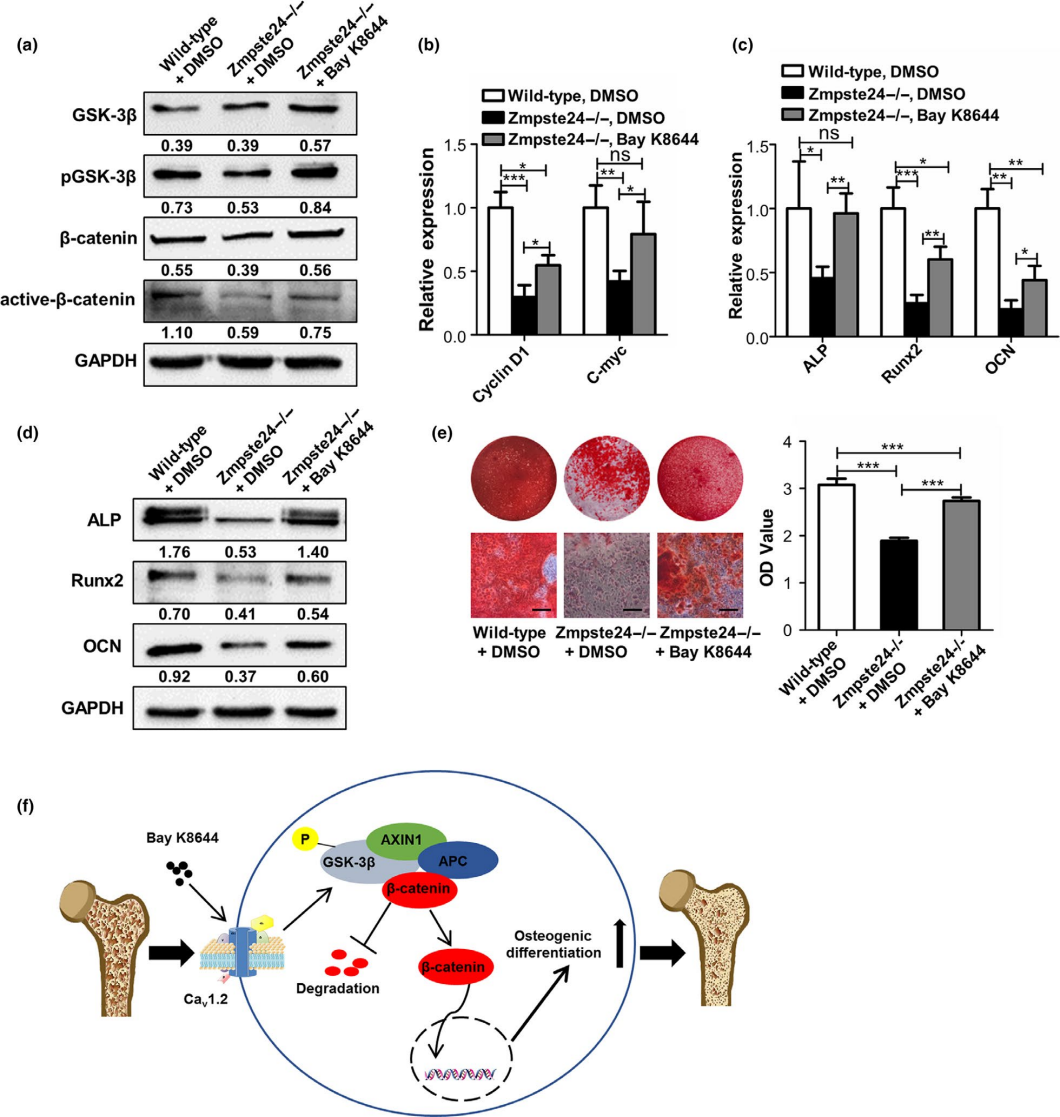
Bay K8644 rescues osteogenic differentiation ability of Zmpste24−/− BMMSCs in vivo. (a) After intraperitoneal injection for 2 months, the protein expression levels of GSK3β, p‐GSK3β, β‐catenin, and active‐β‐catenin of BMMSCs from DMSO‐treated wild‐type, DMSO‐treated Zmpste24−/−, and Bay K8644‐treated Zmpste24−/− mice were confirmed by Western blot analysis (*n* = 6, 7, and 7). (b) After intraperitoneal injection for 2 months, Wnt target genes of cyclin D1 and c‐myc in BMMSCs from DMSO‐treated wild‐type, DMSO‐treated Zmpste24−/−, and Bay K8644‐treated Zmpste24−/− mice were explored by qRT–PCR (*n* = 6, 7, and 7). (c) After intraperitoneal injection for 2 months, expressions of osteogenic‐related genes of *ALP*, *Runx2,* and *OCN* in BMMSCs from DMSO‐treated wild‐type, DMSO‐treated Zmpste24−/−, and Bay K8644‐treated Zmpste24−/− mice were detected by qRT–PCR after osteogenic induction for 5 days (*n* = 6, 7, and 7). (d) After intraperitoneal injection for 2 months, expressions of osteogenic‐related proteins of ALP, Runx2, and OCN in BMMSCs from DMSO‐treated wild‐type, DMSO‐treated Zmpste24−/−, and Bay K8644‐treated Zmpste24−/− mice were detected by Western blot after osteogenic induction for 7 days (*n* = 6, 7, and 7). (e) After intraperitoneal injection for 2 months, alizarin red staining and quantification of BMMSCs from DMSO‐treated wild‐type, DMSO‐treated Zmpste24−/−, and Bay K8644‐treated Zmpste24−/− mice were performed to detect mineralized nodules after osteogenic induction for 14 days (*n* = 6, 7, and 7). (f) Schematic diagram shows that intraperitoneal injection of Bay K8644 improves defective osteogenic differentiation and ameliorates osteoporosis symptom through targeting Ca_v_1.2 channel and canonical Wnt pathway of BMMSCs. The expression levels of the target genes and proteins were normalized to GAPDH. Scale bar, 50 um. Data are shown as mean ± *SD*. *p < 0.05, **p < 0.01, ***p < 0.001, which was determined by unpaired two‐tailed Student's *t* test

After administration of Bay K8644 or DMSO for 2 months, we also assessed its effect on Zmpste24−/− BMMSCs in vivo. Consistent with our expectations, Bay K8644 could activate canonical Wnt pathway of Zmpste24‐deficient BMMSCs, as confirmed by enhanced expressions of p‐GSK3β and active‐β‐catenin (Figure 6a). Besides, Wnt target genes of cyclin D1 and c‐myc were also upregulated in BMMSCs from Bay K8644‐treated Zmpste24−/− mice (Figure 6b). Moreover, as shown by increased levels of osteogenic markers and numbers of mineral node, Bay K8644 could rescue impaired osteogenic differentiation of Zmpste24‐deficient BMMSCs in vivo (Figure 6c–e). To sum up, these results showed that intraperitoneal injection of Bay K8644 improved defective osteogenic differentiation and ameliorated osteoporosis symptom through targeting Ca_v_1.2 channel and canonical Wnt pathway of BMMSCs (Figure 6f).

## 3 DISCUSSION

In this study, we investigated the role of VGCCs played in regulating age‐related bone mass loss and their potential mechanism. We found the bone mass of Zmpste24−/− mice was reduced and the osteogenic differentiation of BMMSCs isolated from Zmpste24−/− mice was impaired. Then we examined expressions of VGCCs in wild‐type and Zmpste24−/− BMMSCs and presented evidence that downregulation of Ca_v_1.2 in Zmpste24−/− BMMSCs contributed to compromised osteogenic capacity through inhibiting Ca_v_1.2 in wild‐type BMMSCs and overexpressing Ca_v_1.2 in Zmpste24−/− BMMSCs. Mechanistically, we elucidated that Ca_v_1.2 regulated osteogenic differentiation ability by targeting Wnt/β‐catenin signaling. We also illuminated that Bay K8644 can be an effective strategy for treating age‐related osteoporosis in virtue of activating Ca_v_1.2 channel and downstream Wnt/β‐catenin signaling and promoting osteogenic differentiation of BMMSCs.

Aging is a gradual deterioration of physical abilities, and osteoporosis is one of its critical features. The molecular basis of osteoporosis during aging remains ambiguous. Compromised osteogenic differentiation abilities of BMMSCs have been demonstrated to be one of key factors contributing to age‐related osteoporosis. Zmpste24‐deficient mice recapitulate many phenotypes observed in physiological aging, and our study also showed the defective bone phenotype of Zmpste24−/− mice and decreased osteogenic differentiation of Zmpste24−/− BMMSCs, which was consistent with previous report (Rivas, Li, Akter, Henderson, & Duque, 2009). It has been borne out by numerous studies that aberrant signal pathways are involved in age‐related bone mass loss, including Notch (Roforth et al., 2014) and Wnt (Garcia‐Velazquez & Arias, 2017). These signaling pathways are intricate with a mixed network. Targeting the upstream regulatory mechanism may yield more effective results in treatment of age‐related bone mass loss. VGCCs are the crucial influence factor of signal pathways and cell function. They encompass several channels with distinct functions (Striessnig, 2016). For example, Ca_v_1.1 can mediate the process of excitation–contraction coupling (Wu et al., 2016), and Ca_v_1.3 is associated with pace‐making in dopaminergic neurons (Singh, Verma, et al., 2016). Abnormity of these channels can lead to a variety of disorders (Zamponi et al., 2015). Numerous studies have confirmed the engagement of VGCCs in age‐related disease, including Parkinson disease (Ca_v_1.3) (Zamponi et al., 2015), ataxia (Ca_v_2.1) (Dorgans et al., 2017), Alzheimer's disease (Ca_v_3.1) (Rice et al., 2014). Despite this, whether and how VGCCs exert influence on osteoporosis remains controversial. Several studies reported that the activation of VGCCs could promote bone formation (Li et al., 2003, 2002; Noh et al., 2011), while others found that inhibition of VGCCs could suppress osteogenesis (Marie, 2010; Ritchie et al., 1994). In this study, we found decreased Ca_v_1.2 expressions in several bone aging models, implying that Ca_v_1.2 is an integral part of intricate mechanisms underlying age‐related osteoporosis.

Ca_v_1.2 is widely expressed in various tissues and regulates multiple cell functions. In term of cell differentiation, it participates in the process of osteogenic (Wen et al., 2012), odontogenic (Ju et al., 2015), and neural differentiation (Obermair, Szabo, Bourinet, & Flucher, 2004). Abnormity of Ca_v_1.2 can lead to several disorders (Zamponi et al., 2015), including Timothy syndrome, cardiac arrhythmias, and neuropsychiatric diseases. Previous study has shown that Ca_v_1.2 can promote osteoblast differentiation and bone formation and rescue estrogen deficiency‐induced osteoporosis (Cao et al., 2017). However, whether Ca_v_1.2 is involved in age‐related osteoporosis remains unknown. In our study, we confirmed Ca_v_1.2 is involved in regulating BMMSCs osteogenic differentiation through gain and loss of function experiments.

Next, we investigated how Ca_v_1.2 participates in regulating defective osteogenic differentiation of aging BMMSCs. It has been widely accepted that canonical Wnt pathway is implicated in the regulation of BMMSCs osteogenic differentiation (Cook et al., 2014; Lin & Hankenson, 2011), and several studies have confirmed the link between calcium channel and Wnt/β‐catenin signaling (Liu et al., 2014; Sagredo et al., 2018). For example, a previous study showed that Wnt/β‐catenin signaling could be regulated by transient receptor potential cation channel subfamily M member 4 (TRPM4) (Sagredo et al., 2018). Thus, we wondered whether Ca_v_1.2 regulated defective osteogenic differentiation of aging BMMSCs through Wnt/β‐catenin signaling. In our study, we observed that canonical Wnt pathway was decreased in Zmpste24−/− BMMSCs and improving this signal pathway could ameliorate impaired osteogenic differentiation of aging BMMSCs. These results suggested that impaired osteogenic differentiation of BMMSCs was, to some degree, due to decreased canonical Wnt pathway. We also overexpressed Ca_v_1.2 in Zmpste24−/− BMMSCs in the context of siRNA‐mediated β‐catenin silencing followed by analysis of the osteogenic differentiation, and the results further confirmed the involvement of canonical Wnt pathway for Ca_v_1.2‐mediated osteogenic differentiation.

Since Ca_v_1.2 belongs to L‐type calcium channel and Bay K8644 is regarded as a potent L‐type Ca^2+^ channel agonist, we speculated if Bay K8644 is capable of rescuing osteogenic differentiation abnormality through activating Ca_v_1.2 channel. Treatment of Zmpste24−/− BMMSCs with Bay K8644 at the concentration of 10^‐7 ^M could promote canonical Wnt pathway and relieve osteogenic differentiation deficiency. Through using Ca_v_1.2 siRNA during Bay K8644 treatment, we further confirmed Bay K8644 improved osteogenic differentiation of Zmpste24−/− BMMSCs through Ca_v_1.2 channel. These data suggested that osteogenic differentiation decline in Zmpste24−/− BMMSCs can be rescued by Bay K8644, which is attributable largely to the activation of Ca_v_1.2 channel and downstream Wnt/β‐catenin pathway.

We further demonstrated the feasibility of Bay K8644 to treat age‐related bone loss. However, despite the results that osteogenic differentiation deficiency and bone mass loss being improved upon receiving Bay K8644 stimulation, this method had limited effects as results from micro‐CT and double‐calcein labeling only showed partial recovery of osteoporosis features after Bay K8644 treatment. We ascribed it to the complex etiology of age‐related bone mass loss as it is a disease involving multiple factors, of which defective osteogenic differentiation is just one element (Fossett, Khan, Pastides, & Adesida, 2012; Singh, Brennan, et al., 2016; Wang et al., 2014). In terms of Zmpste24‐deficient mice, age‐related features mainly result from devastation of nuclear envelope and accumulation of progerin (Lee et al., 2016). Therefore, looking for another upstream mechanism for age‐related bone mass loss will be the focus of our future research on treating age‐related bone mass loss.

In summary, we indicate that defective osteogenic differentiation of Zmpste24−/− BMMSCs can be partly attributed to the inhibition of canonical Wnt pathway which is partly owing to decreased Ca_v_1.2 expression, and Bay K8644 treatment could be an applicable approach for treating age‐related bone loss by ameliorating compromised osteogenic differentiation capacity through targeting Ca_v_1.2 channel and canonical Wnt pathway. Taken together, we propose a new mechanistic explanation underlying age‐related bone loss and propose a feasible strategy via Ca_v_1.2 and Wnt/β‐catenin pathway activation.

## 4 EXPERIMENTAL PROCEDURES

### 4.1 Animal

Animal experiments were approved by Fourth Military Medical University and performed in accordance with the committee guidelines of Intramural Animal Use and Care Committee of Fourth Military Medical University. Zmpste24−/− mice of C57BL/6J strains were offered by Professor Zhongjun Zhou from University of Hong Kong, which have been described previously (Pendas et al., 2002), and the wild‐type mice were obtained from the breeding of Zmpste24+/‐ mice. Male littermates were used all through the study. Genotype was identified via real‐time polymerase chain reaction, and the primers are listed in Table S2.

### 4.2 Cell culture

Primary BMMSCs were isolated as previously described (Liao et al., 2016). Briefly, BMMSCs from 3‐month‐old mice were obtained and cultured in α‐MEM (Gibco BRL) supplemented with 20% fetal bovine serum (FBS; Thermo Electron), 0.292 mg/ml glutamine, 100 U/ml penicillin, and 100 mg/ml streptomycin (Invitrogen). Cells were then plated in the 10 cm dish (Costar) at 37°C in 5% CO_2,_ and the medium was changed every 3 days. BMMSCs at passage 1 were used in the experiments. Bay K8644 (Abcam) was dissolved in DMSO (0.1% culture concentration).

### 4.3 Osteogenic differentiation

Before osteogenic induction, 2 × 10^5^ BMMSCs were seeded on 12‐well culture dishes and cultured in the growth medium until the cells reached 80%–90% confluence as defined by area covered. BMMSCs were then cultured under osteogenic culture conditions containing 100 μg/ml ascorbic acid (Sigma), 5 mmol/l β‐glycerophosphate (Sigma), and 10 nmol/l dexamethasone (Sigma). After osteogenic induction for 5 days, quantitative RT–PCR (qRT–PCR) was performed for the expressions of alkaline phosphatase (ALP), runt‐related transcription factor 2 (Runx2), and osteocalcin (OCN). Osteogenesis related proteins of ALP, Runx2, and OCN were assayed by Western blot after osteogenic induction for 7 days. Alizarin red staining was used to assess calcium deposit on day 14, and 10% cetylpyridinium chloride was added for quantitative analysis. The absorbance values were measured at 562 nm.

### 4.4 Transfection assays

BMMSCs were seeded on twelve‐well culture dishes and grown to 80%–90% confluence followed by serum starvation for 2h. Ca_v_1.2 (Santa Cruz Biotechnology, sc‐42689) or β‐catenin siRNA (Santa Cruz Biotechnology, sc‐29210) was transfected into BMMSCs at a final concentration of 50 nM, and overexpression plasmid of Ca_v_1.2 (addgene, Plasmid #26572) was transfected into BMMSCs at 500 ng. For the control groups of siRNA or plasmid transfection experiments, scramble siRNA (RiboBio) or control overexpression vector pcDNA6/V5‐His (Invitrogen) was included. Lipo2000 (Invitrogen) was used as a transfection reagent according to the manufacturer's instructions. After transfection, the culture medium was substituted by normal culture medium and cells were harvested at 48 hr for RNA and 72 hr for protein extraction. For detection of the osteogenic differentiation capacity, transfection medium was removed in the next day and replaced by osteogenic induction medium.

### 4.5 Quantitative RT–PCR

Total cellular RNA was extracted from cells using the TRIzol reagent™ (Invitrogen) according to the manufacturer's instructions and cDNA was synthesized in a 20 μl reaction volume (Takara). The qRT–PCR reactions were performed in a total volume of 10 μl using the SYBR Premix Ex Taq™II kit (Takara) and then detected on the CFX96 Real‐Time System (Bio‐Rad). Fold changes of mRNA were calculated by the $2^{-\Delta \Delta C_{\text{t}}}$ method after normalization to the expression of GAPDH. The primer set sequences used for this study are listed in Table S3.

### 4.6 Western blot analysis

Cells were washed with PBS twice and lysed in RIPA lysis buffer (Beyotime) at 4°C for 2 hr. Samples were separated using 10% Tris‐glycine SDS‐polyacrylamide gel (Invitrogen) and transferred to PVDF membranes with a current of 200 mA for 2 hr. After blocked with 5% albumin from bovine serum (BSA) in PBST (PBS with 0.1% Tween), membranes were incubated overnight at 4°C with the following primary antibodies: anti‐GAPDH (CWBIO, CW0100), anti‐ALP (Abcam, ab108337), anti‐Runx2 (Cell Signaling Technology, #12556), anti‐OCN (Santa Cruz Biotechnology, sc‐390877), anti‐GSK3β (Cell Signaling Technology, #9832), anti‐β‐Catenin (Cell Signaling Technology, #8480), anti‐phospho‐GSK3β (Cell Signaling Technology, #9323), anti‐active‐β‐Catenin (Millipore, 05‐665), and anti‐Ca_v_1.2 (Alomone labs, Acc‐003). Then, the protein bands were incubated with secondary antibody (Jackson) and visualized using an enhanced chemiluminescence kit (Pierce) according to the manufacturer's instructions. The quantitative data of Western blot were analyzed by Image J (National Institutes of Health).

### 4.7 TOP/FOP flash reporter assay

1 × 10^5^ 293T cells were seeded into 24‐well culture dishes and grown to 80%–90% confluence followed by serum starvation for 2 hr. To detect the activity of β‐catenin after Ca_v_1.2 overexpression, cells were transfected with progerin (OBiO Technology) and pcDNA6/V5‐His plasmids in the control groups and progerin and Ca_v_1.2 plasmids in the experimental groups. To detect the activity of β‐catenin after 10^−7^ M Bay K8644 treatment, cells were transfected with progerin plasmid and stimulated with DMSO in the control groups and progerin plasmid and stimulated with Bay K8644 in the experimental groups. After 48 hr, the cells were co‐transfected with 0.25 μg TOP (Millipore) containing the TCF/LEF consensus sequence or 0.25 μg FOP (Millipore) containing mutated TCF binding sites flash luciferase reporter vector and 0.05ug Renilla luciferase plasmid pRL‐SV40 (Promega). Lipo2000 was used as a transfection reagent. 24 hr later, the luciferase assay was performed to detect the activity of β‐catenin by using dual‐luciferase reporter assay kit (Promega, #E1910). GloMax 20/20 (Promega) was used to detect Firefly or Renilla luciferase activity.

### 4.8 MTT assay

For the detection of cell proliferation, 1 × 10^4^ Zmpste24−/− BMMSCs were seeded on ninety‐six‐well culture dishes. One day later (day 1), the culture medium was moved, and growth medium containing DMSO, 10^−6^ M, 10^−7^ M, and 10^−8^ M Bay K8644 were added to the wells, respectively. MTT assay was performed on day 4. The process was as followed: First, 20 μl MTT (5 mg/ml in PBS) was added to the well; second, after culturing for 4 hr, the medium was carefully moved and 150 μl DMSO was added; and third, after vibrating for 10 min, the quantitative analysis was performed with 490 nm absorbance values.

### 4.9 Intracellular calcium concentration analysis

For the detection of intracellular calcium concentration, BMMSCs were incubated with 5 μM Fluo‐3/AM dye (Invitrogen, Life Technology) for 60 min at 37°C, followed by washing with PBS for three times. After digestion by trypsin, 2 × 10^5^ cells were counted with PBS washing for two times. Then, cells were subjected to flow cytometric analysis (Beckman Coulter). Data are presented as fluorescence intensity.

### 4.10 Calcium imaging

For the calcium imaging, BMMSCs were incubated with 5 μM Fluo‐3/AM dye for 30 min at 37°C, followed by washing with calibrated EGTA/Ca^2+^ solutions for three times. Images were collected every 4 s at 2 Hz with excitation at 488 nm and emission at 530 nm by confocal laser microscopy (Zeiss, Oberkochen FV1000). 30 mM KCl was added to the culture medium at 60 s. Fluo‐3 fluorescence intensity reflected intracellular Ca^2+^ level, which was described previously (Merritt, McCarthy, Davies, & Moores, 1990). The formula of Fluo‐3 fluorescence intensity increase ratio is (*F*–*F*_0_)/*F*_0_. *F* means the fluorescence value detected and *F*_0_ represents the minimum fluorescence value.

### 4.11 Micro‐CT analysis

To assess bone mass in mice, micro‐CT (eXplore Locus SP, GE Healthcare) was used to scan femora. After sacrifice, bones were isolated and fixed in 4% paraformaldehyde overnight. The distal femoral metaphysis was scanned at a voltage of 80 kV with a current of 80 μA, and the voxel‐size (mm) was (0.015898 × 0.015898 × 0.015898). The region of the trabecular bone for analyzing was defined from 0.1 to 1.5 mm away from the epiphyseal growth plate. The trabecular bone from each selected slice was segmented for three‐dimension reconstruction. Parameters of BMD, BV/TV, and Tb.N were analyzed with the Micview software.

### 4.12 Calcein labeling assay

Mice were intraperitoneal injected with 20 mg/kg calcein (Sigma) at day 14 and day 2 before sacrifice. The femora were isolated and fixed in 80% ethanol at 4°C for 48 hr. After using graded ethanol to dehydrate, the femora were embedded in methyl methacrylate and longitudinal cut into 30 μm thick sections using a hard tissue slicing machine (SP1600, Leica). Bone dynamic histomorphometric analyses for mineral apposition rate (MAR) were performed according to the standardized nomenclature for bone histomorphometry under fluorescence microscopy (Leica DM 6000B, German).

### 4.13 Statistical analyses

All experiments were repeated at least three times, and data are presented as mean ± *SD*. Comparisons between two groups were performed using paired or unpaired two‐tailed Student's *t* test. SPSS13.0 software was utilized, and a value of *p* < 0.05 was considered statistically significant (*p < 0.05; **p < 0.01; ***p < 0.001).

## CONFLICT OF INTEREST

None declared.

## AUTHOR CONTRIBUTIONS

Bei Li, Qintao Wang, and Yan Jin designed and supported the experiments. Dongdong Fei, Yang Zhang, Junjie Wu, and Hui Zhang performed the experiments and analyzed the data. Bei Li, Dongdong Fei, and Anqi Liu wrote the manuscript. Xiaoning He and Jinjin Wang provided helpful discussion and technical support. All authors have reviewed and agreed the final version of the manuscript.
