# Supplementary material for: Cav1.2 regulates osteogenesis of bone marrow‐derived mesenchymal stem cells via canonical Wnt pathway in age‐related osteoporosis
Source: Aging Cell. 2019 May 23;18(4):e12967. doi: 10.1111/acel.12967 (PMC6612635; doi:10.1111/acel.12967)
Supplement: Supplementary file 1 [file ACEL-18-e12967-s001.pdf]

Supplementary data

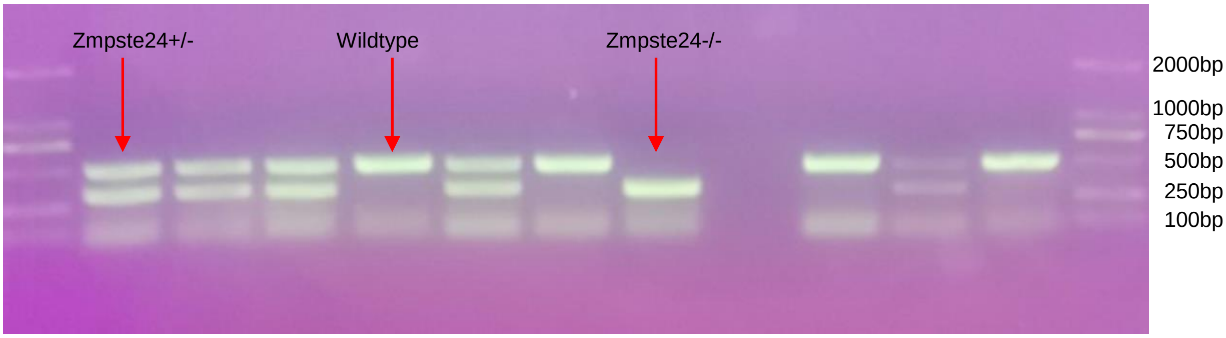

Fig. S1 Genotype of the wild-type, Zmpste24+/- and Zmpste24-/- mice.

Genotype was identified via real-time polymerase chain reaction, and the primers are listed in Supplementary Table 2.

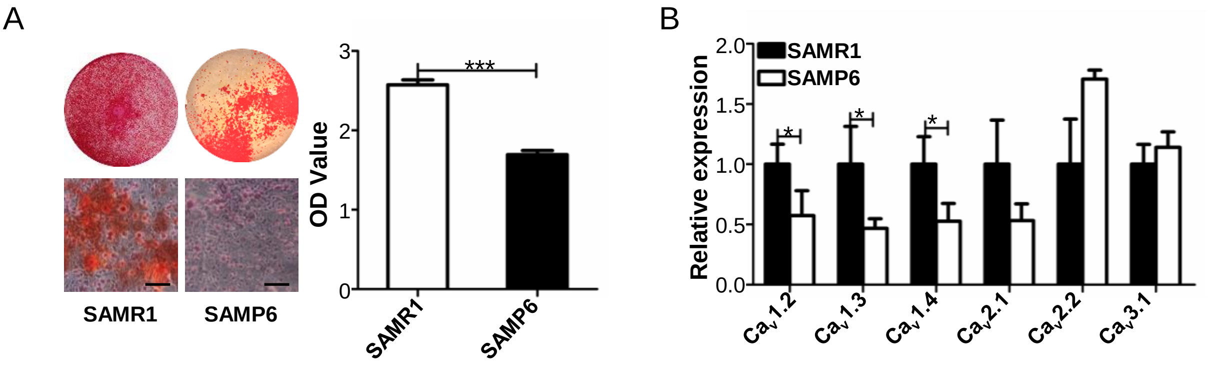

Fig. S2 Ca_v1.2 mRNA expression is lower in BMMSCs from SAMP6 than SAMR1.

(A) 3-month-old SAMR1 and SAMP6 that obtained from the Council for SAM Research of Kyoto University were used, and passages 1 of BMMSCs were used for this study. Alizarin red staining was performed to detect mineralized nodules (n=3). Scale bar, 50 um. (B) Expressions of VGCCs related genes in BMMSCs from SAMP6 and SAMR1 were detected by qRT-PCR (n=5). The expression levels of the target genes were normalized to GAPDH. The primers are listed in Supplementary Table 3. Data are shown as mean ± SD. *P<0.05, **P<0.01, ***P<0.001, which was determined by unpaired two-tailed Student's t-test. SAMR1: senescence-accelerated mice-resistant 1; SAMP6: senescence-accelerated mouse prone 6.

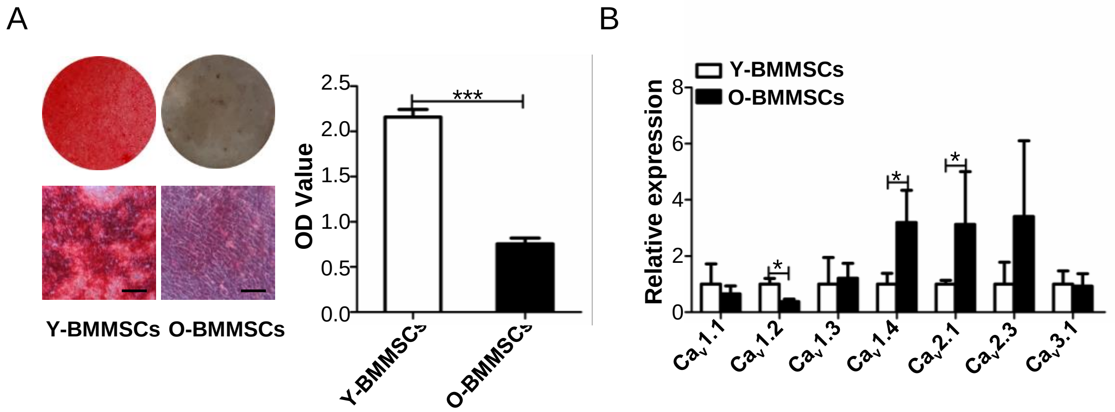

Fig. S3 $Ca_v1.2$ mRNA expression is downregulated in BMMSCs from old individuals (O-BMMSCs) than young individuals (Y-BMMSCs).

Human BMMSCs were collected from the First Hospital Affiliated to the Fourth Military Medical University and the donor information was listed in Supplementary Table 1. The use of human BMMSCs in this study was approved by the Ethics Committee of Fourth Military Medical University. Human bone marrow samples aspirated from iliac were diluted with equal volume of Dulbecco‘s phosphate buffered saline (DPBS), followed by centrifugation at 2000 rpm for 30 min. Then, the cells were plated in a culture plate and cultured in α-MEM supplemented with 10% fetal bovine serum. Passages 3-6 of cells were used for this study. (A) Alizarin red staining was used to assess calcium nodules deposit on day 28, and 10% cetylpyridinium chloride was added for quantitative analysis. The absorbance values were measured at 562 nm (n=5). Scale bar, 50 um. (B) Expressions of VGCCs related genes from Y-BMMSCs and O-BMMSCs were detected by qRT-PCR (n=5). The expression levels of the target genes were normalized to GAPDH. The primers are listed in Supplementary Table 3. Data are shown as mean ± SD. *P<0.05, **P<0.01, ***P<0.001, which was determined by unpaired two-tailed Student’s t-test. Y-BMMSCs: BMMSCs from young individuals; O-BMMSCs: BMMSCs from old individuals.

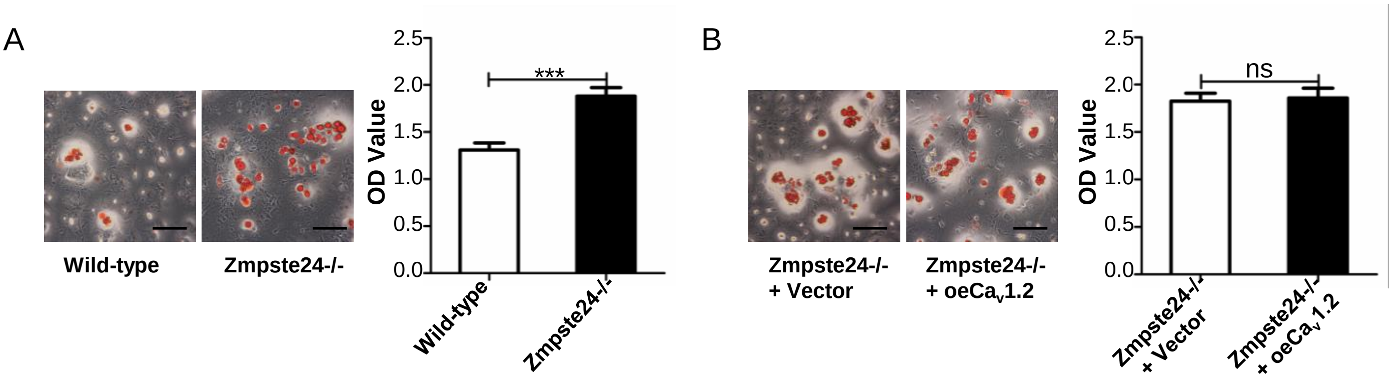

Fig. S4 Ca$_v$1.2 has a limited effect on adipogenic differentiation of Zmpste24-/- BMMSCs. (A) BMMSCs from wild-type and Zmpste24-/- mice were cultured under adipogenic culture conditions containing 10 μg/ml insulin (Sigma-Aldrich), 500 nM isobutylm ethylxanthine (MP Biomedicals, Irvine, CA, USA), 60 μM indomethacin (Sigma-Aldrich), 500 nM hydrocortisone (MP Biomedicals, Irvine, CA, USA), and 100 nM L-ascorbicacid phosphate. After adipogenic induction for 5 days, the cultured cells were stained with Oil Red-O (Sigma-Aldrich), and 100% isopropanol was added for quantitative analysis (n=3). The absorbance values were measured at 490 nm. ***P<0.001 (unpaired two-tailed Student's t-test). (B) Zmpste24-/- BMMSCs were transfected with control overexpression vector or Ca$_v$1.2 vector plasmid. After adipogenic induction for 5 days, Oil red O staining and quantification of Zmpste24-/- and Ca$_v$1.2-overexpressed Zmpste24-/- BMMSCs were performed to detect lipid droplets (n=3), and paired two-tailed Student's t-test was used. Scale bar, 50 um. Data are shown as mean ± SD. ***P<0.001, which was determined by unpaired two-tailed Student's t-test.

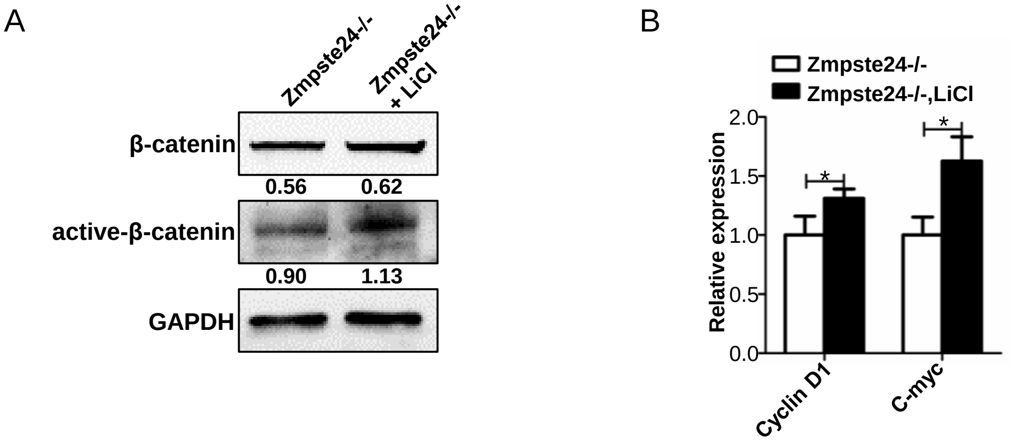

Fig. S5 Lithium chloride (LiCl) activates Wnt/β-catenin pathway. (A) Zmpste24-/- BMMSCs were stimulated by 5 mM lithium chloride (Sigma, USA), and the expressions of active-β-catenin in non-treated and treated cells were explored by western blot (n=3). (B) Zmpste24-/- BMMSCs were stimulated by 5 mM lithium chloride, and Wnt target genes of cyclin D1 and c-myc in non-treated and treated cells were explored by qRT-PCR (n=3). *P<0.05 (paired two-tailed Student's t-test). Data are shown as mean ± SD.

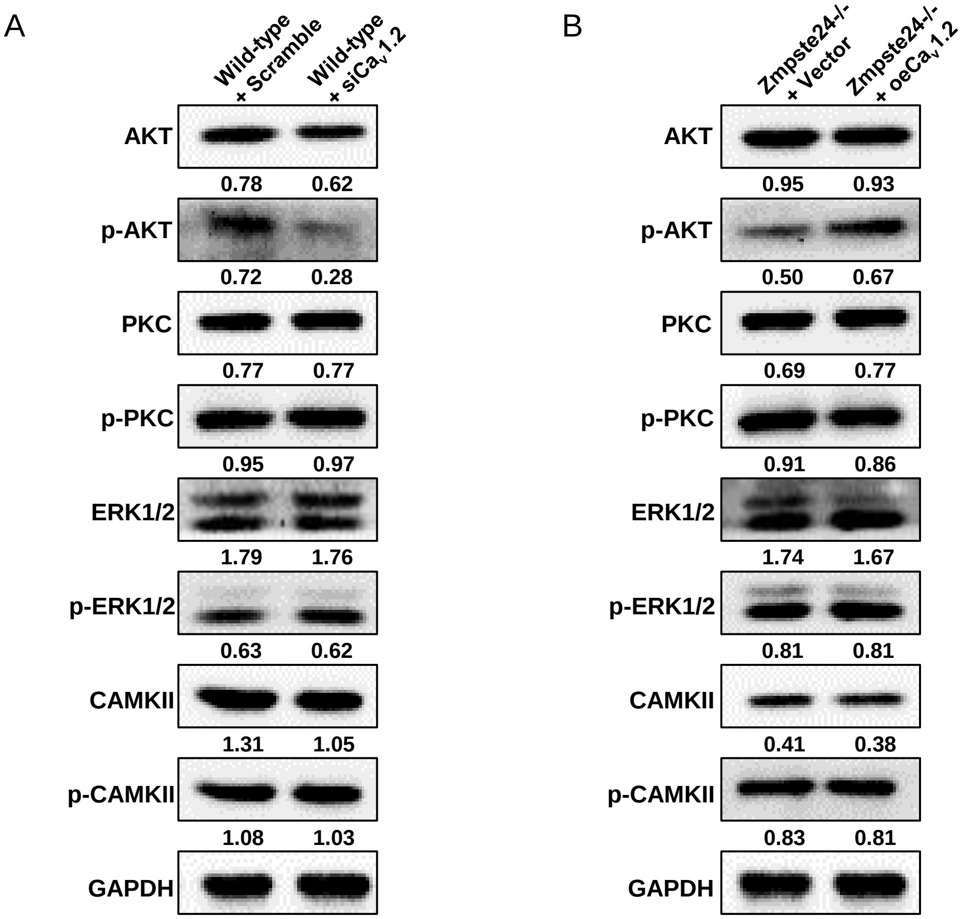

Fig. S6 The effect of Ca$_v$1.2 on the downstream signaling pathway of calcium current was explored. (A) Wild-type BMMSCs were transfected with scramble siRNA or Ca$_v$1.2 siRNA, the protein expression levels of AKT, p-AKT, PKC, p-PKC, ERK1/2, p-ERK1/2, CAMKII and p-CAMKII were measured by western blot analysis after 72 h (n=3). (B) Zmpste24-/- BMMSCs were transfected with control overexpression vector or Ca$_v$1.2 vector plasmid, the expression levels of associated proteins were measured by western blot analysis after 72 h (n=3). Antibodies to mouse phospho-Erk1/2 (Thr202/Tyr204), Erk1/2, phospho-AKT, phospho-PKC, PKC, phospho-CamkII, AKT were purchased from Abcam (Cambridge, UK). Antibodies to mouse CamkII was obtained from Millipore (Billerica, MA, USA).

Supplementary tables

Supplementary Table. 1 Donor information of human BMMSCs

Cells	Age	Gender
Y-BMMSCs1	7	male
Y-BMMSCs2	19	male
Y-BMMSCs3	21	male
Y-BMMSCs4	22	male
Y-BMMSCs5	24	female
O-BMMSCs1	55	missing
O-BMMSCs2	57	female
O-BMMSCs3	60	male
O-BMMSCs4	62	male
O-BMMSCs5	75	male

Supplementary Table. 2 Sequence of genotypic identification

primer	Forward (5’-3’)	Reverse (5’-3’)
m-wildtype	CTAGTCAGCTGGCCTTGTTGCTG	GCCAACCTAGACTCTGCCAGGAT
m-mutant	CTAGTCAGCTGGCCTTGTTGCTG	GGAGCGGATCTCAAACTCTCCTC

Supplementary Table. 3 Sequence of primers for qRT-PCR

mRNA/primer	Forward (5'-3')	Reverse (5'-3')
m-GAPDH	TGTGTCCGTCGTGGATCTGA	TTGCTGTTGAAGTCGCAGGAG
m-Runx2	CCGCACGACAACCGCACCAT	CGCTCCGGCCCACAATCTC
m-ALP	CCTTGTAGCCAGGCCCATTG	GGACCATTCCCACGTCTTCAC
m-OCN	GGACCATCTTTCTGCTCACTC	CACTACCTTATTGCCCTCCTG
m-Ca_v1.1	GAACCTTCCAAAAGCTCC	CAAGTCGTCCACGTCCATGAC
m-Ca_v1.2	TGCCTTCAAACCCAAGCACT	GGTGTACCTCGGTGATTGCTATATC
m-Ca_v1.3	TTTGACAATGTCCTTTCGGCT	TTCTCACCGTTTGAATCAATAGCT
m-Ca_v1.4	CTTCATCTATGCAGTCATTGGCA	CCTGCGGAAAGGTCTGGAA
m-Ca_v2.1	CATCATCATCGGCTCCTTTT	GAAAAGCTCTCCGGTTCTCC
m-Ca_v2.2	GAAAAGCTCTCCGGTTCTCC	TTTAGGCAGCCGCTTGATG
m-Ca_v2.3	AACCCACTTCAACACCCACG	GCACGATCTGCAGGCTAGGT
m-Ca_v3.1	CGCTGACCATGAAATGCC	GCCGTCGCCGTCTGTG
m-Ca_v3.2	TGGTTCGAGCACATTAGCATG	CTGCAACGTTCTGAACGGC
m-Ca_v3.3	GGGCATCAGTGGCTGTAGTT	GTGCACCCTGAATTGCTTCT
m-cyclin D1	GCAAGCATGCACAGACCTTT	GCAGTCCGGGTCACACTTGA
m-c-myc	CCTAGTGCTGCATGAGGAGAC	CGTAGTTGTGCTGGTGAGTG
h-GAPDH	GCACCGTCAAGGCTGAGAAC	TGGTGAAGACGCCAGTGGA
h-Runx2	CGGAATGCCTCTGCTGTTATG	AAGGTGAAACTCTTGCCTCGTC
h-ALP	GGACCATTCCCACGTCTTCAC	ATGACAGTACCGCCCATTGC
h-OCN	GCCACCGAGACACCATGAGA	AGGCTGCACCTTTGCTGGAC
h-Ca_v1.1	CTCCAGCGGGGGACTGTATT	CACCAAGGCGATCTTCCCAA
h-Ca_v1.2	GCCGAAGACATCGATCCTGA	GAAAATCACCAGCCAGTAGAAGA
h-Ca_v1.3	GCATTGGGAACCTTGAGCATGTGTCTG	GCGAGCTGTCATCCTCGTAGC
h-Ca_v1.4	CGGAATCTGAAGGCGGGAAA	CCACTGCTTTCACCTTCCAC
h-Ca_v2.1	CGATGCCTCAGGGAACACTTGG	CCATGTACCCATTGAGCTCACG
h-Ca_v2.2	CATCAACCGCCACAACAACT	ACATCAGAAAGGAGCACAGG
h-Ca_v2.3	TCGCTGTGGATAATCTCGCC	CTCCCTCAGGTGGCTGCT
h-Ca_v3.1	GGTCCGGCACAAGTACAACT	CACAATGAGCAGGAACGAGA
h-Ca_v3.2	TCGAGGAGGACTTCCACAAG	TGCATCCAGGAATGGTGAG
h-Ca_v3.3	GGTGTCGTGGTGGAGAACTT	GTGCACATGGAGTGGATGAG
